# *QuickStats:* Death Rates for Drug Overdose* Among Persons Aged 25–44 Years, by Race and Ethnicity^†^— United States, 2000–2020

**DOI:** 10.15585/mmwr.mm7146a4

**Published:** 2022-11-18

**Figure:**
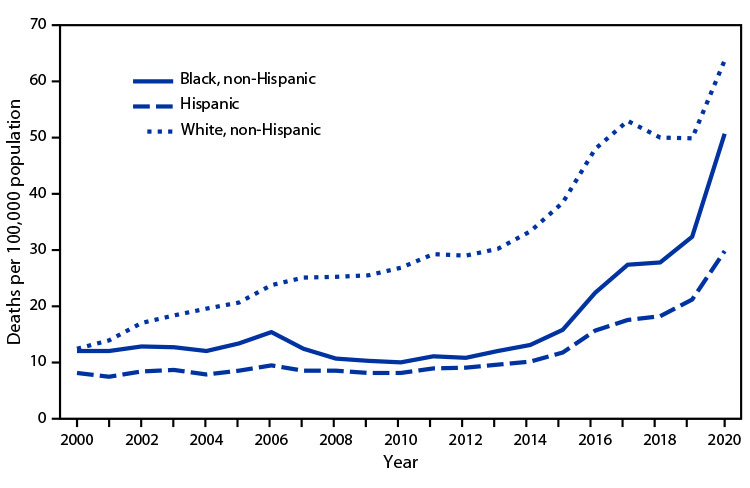
From 2000 to 2013, the rate for drug overdose death increased for non-Hispanic White (White) persons aged 25–44 years but was stable for non-Hispanic Black (Black) and Hispanic persons in this age group. From 2013 to 2020, rates increased for all groups, from 30.2 to 63.8 per 100,000 population for White persons, from 12.0 to 50.7 for Black persons, and from 9.6 to 29.9 for Hispanic persons. From 2019 to 2020, all three racial and ethnic groups experienced the largest annual increase in drug overdose death rates (56% among Black, 41% among Hispanic, and 28% among White persons). In 2020, the drug overdose death rate for White persons was the highest among all groups, followed by Black and Hispanic persons.

**Source:** National Vital Statistics System, Mortality Data. https://www.cdc.gov/nchs/nvss/deaths.htm

For more information on this topic, CDC recommends the following link: https://www.cdc.gov/drugoverdose/health-equity/index.html

